# Microbiota, Intestinal Immunity, and Mouse Bustle

**Published:** 2014

**Authors:** A. A. Kruglov, S. A. Nedospasov

**Affiliations:** Belozersky Research Institute of Physico-Chemical Biology and Department of Immunology, Biological Faculty, Lomonosov Moscow State University, Leninskie Gory, 1, 119991, Moscow, Russia; Engelhardt Institute of Molecular Biology, Russian Academy of Sciences, Vavilova Str., 32, 119991, Moscow, Russia; German Rheumatism Research Center, Chariteplatz 1, 10117, Berlin, Germany

## Abstract

The composition of the intestinal microbiota is regulated by the immune system. This paper discusses the role of cytokines and innate immunity lymphoid cells in the intestinal immune regulation by means of IgA.

## ARTICLE

Our gut is filled with quadrillions of symbionts, commensal bacteria that fulfill, as demonstrated by Honda and Littman [1], additional functions useful to the host. The discovery of the mechanisms that regulate innate immunity has brought forth a surprising riddle: how does the immune system establish a balance in the intestines that does not allow an inflammatory response to develop. Indeed, pattern recognition receptors on the cells of innate immunity, such as macrophages and dendritic cells, recognize commensal bacteria in the same way as they recognize opportunistic pathogenic and pathogenic bacteria. As a result of such recognition, defense reactions are triggered that may be dangerous to the host.

Another aspect of the problem relates to the use of antibiotics, because “good” bacteria respond to antibiotics in about the same way as pathogenic ones do. Recently, Ubeda *et al *[2] demonstrated convincingly that, upon systemic antibiotic therapy, undesirable pathophysiological reactions may develop in model organisms, up to the formation of neoplasia. A new concept has emerged, holding that commensal bacteria are “tuning” the immune system in the gut, providing it with a “tonic” signal.

Analysis of the diversity of commensal organisms in the human gut and in experimental animals (microbiota) conducted using new technologies over recent years has led to the understanding that not only a predisposition to various diseases, but also the response to the therapy depend on microbiota composition.

**Fig. 1:**
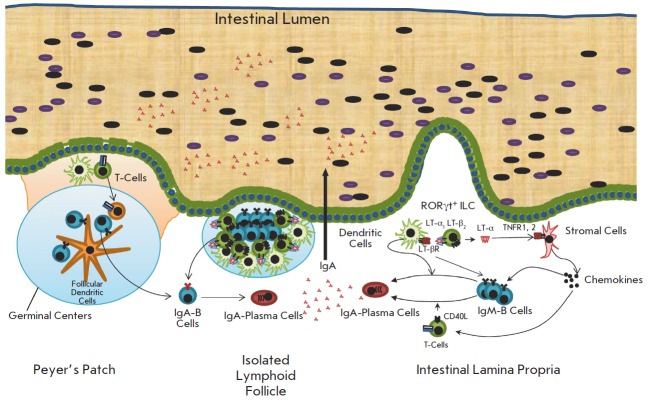
The lymphoid system in the small intestine and a scheme of the IgA production. The switch to IgA can be induced in the Peyer’s patches, isolated lymphoid follicles, and in lamina propria. In the lamina propria, IgA induction is controlled by LT-α and LT-β, which are produced by type III innate lymphoid cells

Intestinal immunity is provided by the same tools that the immune system generally has. These include lymphoid organs (Peyer’s patches and lymph nodes draining the intestine), the arsenal of innate immune cells (part of which perform regulatory functions), and lymphocytes that, in particular, produce protective antibodies (*Fig. 1*).

Antibodies, primarily IgA, participate in both the protection of the gut and in the regulation of the intestinal microbiota composition. For their production, B-lymphocytes, which initially express membrane-bound antibodies of the IgM type, need to first reach the gut compartments associated with immune responses and, then, “switch,” under the influence of the microenvironment and soluble factors, to the production of IgA and to become plasma cells (*Fig. 1*). Several mechanisms responsible for the recruitment of B-lymphocytes into the intestinal lamina propria and for their switching to IgA production are known.

Unique mice were previously generated in our laboratory and used to study the mechanisms controlling the production of IgA antibodies in the intestine [3]. These models employed the methods of “reverse genetics,” in particular, the so-called “conditional knockout” in mice. Such technology is based on manipulations with embryonic stem cells and, for mammals, was developed only for rodents, such as mice and (very recently) rats. This explains why most of the information about immunity mechanisms has been obtained primarily in mice.

By using the conditional knockout technology, we generated unique mice that differed from wild type mict by defects in cytokine expression in different types of cells of both innate and adaptive immunity. If the phenotypic difference, such as functional defects in the intestinal immune system are observed in these mice, then the function of a certain cytokine produced by a specific cell type can be deduced.

One of the areas of our research interests is the cytokines of the tumor necrosis factor (TN F) family; in particular, the lymphotoxins (LT) α and β. These two molecules form a single membrane complex. It was, therefore, believed that most of the physiological functions of LTα and LTβ coincide, because the signal is transmitted through a single receptor, the LT-β receptor (TN FRSF3). At the same time, lymphotoxin-α can occur in soluble trimeric form. In this case, it is very similar to the classic TN F and uses its receptors (p55 and p75). To date, separate, unique (non-redundant) functions of soluble LT-α *in vivo *have not been demonstrated: so the TN F-like properties of this cytokine *in vitro *have been perceived as a curiosity.

However, when we “turned off” the LT-α and LT-β genes in type III innate lymphoid cells (ILC3), which are characterized by the expression of the RORyt transcription factor, we detected differences that allowed us to suggest a new mechanism for immunity regulation in the gut.

**Fig. 2:**
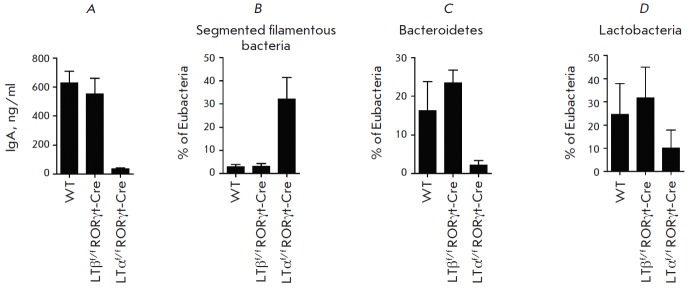
The role of LT-α and LT-β, produced by type III innate lymphoid cells, in the control of IgA and in regulation of microbiota composition. A - the IgA content in the feces of wild type (WT) mice and mice with deletion of the gene LT-α (LTα^f/f^RORγt-Cre) and LT-β (LTβ^f/f^RORγt-Cre) in type III innate lymphoid cells. B - fraction of segmented filamentous bacteria, C – Bacteroidetes, and D - Lactobacteria in the contents of the terminal ileum of WT mice, LTα^f/f^RORγt-Cre, and LTβ^f/f^RORγt-Cre

It turns out that, on one hand, the LT-α/LT-β membrane complex, when transmitting a signal from ILC to stromal and dendritic cells, regulates the recruitment of B-lymphocytes of the B1 and B2 types to lamina propria and triggers the controls antibody isotype switch through a special mechanism in which reactive nitrogen species play an important role (*Fig. 1*). On the other hand, soluble trimeric LT-α, acting through the TN F receptors, recruits not only B-, but also T-lymphocytes, and these are T-lymphocytes that regulate the switch from IgM to IgA and that express a ligand for the CD40 receptor of B-lymphocytes (*Fig. 1*), (*Fig. 2*).

Thus, we stumbled on the function of LT-α (different from LT-β)* in vivo*, and this paradoxical TN Flike function of soluble trimeric LT-α is associated with the regulation of IgA production in the intestines and with microbiota composition control (*Fig. 2*).

An important clinical aspect of our study that needs further investigation is the fact that one of the most popular therapeutic TN F inhibitors, etanercept (Enbrel), which is already being used in millions of patients with rheumatoid arthritis, blocks soluble LT- α. Before our study, it had been believed that this cytokine has no individual functions, and so its inhibition had not been considered as a potential source of side effects.

Interestingly, the only known type of autoimmune diseases, when all TN F inhibitors, except for etanercept, are effective, is actually intestinal inflammatory pathologies [4]. An explanation of this paradox has yet to be provided...
